# The prevalence of Martin-Gruber anastomosis in Iranian subjects by electrodiagnostic criteria

**Published:** 2015-10-07

**Authors:** Saeed Khosrawi, Lida Kianimehr, Somayeh Andalib

**Affiliations:** ^1^ Department of Physical Medicine and Rehabilitation, School of Medicine, Isfahan University of Medical Sciences, Isfahan, Iran

**Keywords:** Martin-Gruber Anastomosis, Electrodiagnostic Criteria, Prevalence, Iran

## Introduction

Median–ulnar anastomosis [Martin–Gruber anastomosis (MGA)] is a common anatomic variant. The crossover often occurs in mid-forearm. Median fibers that have crossed then run with distal ulnar nerve to innervate any of following ulnar muscle: (i) Abductor digiti minimi (ADM), (ii) first dorsal interosseous (FDI), (iii) adductor pollicis, (iv) deep head of flexor pollicis brevis, or (v) combination of these. The FDI is the most common termination (34%), followed by hypothenar (15.5%) and then Thenar (12%) musculature.^1^ This anomaly is asymptomatic. It seems to have less prevalence in our population, however, we did not find any published document regarding the prevalence of this anomaly, so the main goal of this study was to estimate the frequency of MGA in referred subjects to academic electrodiagnostic (EDX) clinics of physical and rehabilitation (PM and R) department.

This descriptive cross sectional study was performed in subjects referred to our EDX clinics of PM and R department. Ninety subjects were recruited who had normal neurological exam. The subjects with median or ulnar nerves injuries due to trauma or polyneuropathy were excluded by supra max stimulate at wrist and elbow. The median nerve compound muscle action potential (CMAP’s) was recorded from abductor pollicis brevis (APB), ADM, and FDI. Afterward, the ulnar nerve was stimulated at wrist and below elbow recording from abductor ADM and FDI and APB muscles. The examinations were conducted in both sides.

After recording these CMAP’s, four conditions may have occurred: (1) All the tests were within normal range; (2) while stimulating ulnar nerve and recording from FDI, > 20% decline in amplitude between wrist and elbow was detected and recording from ADM was normal, then median was stimulated in antecubital fossa whereas recording from FDI; provided that MGA was present, the amplitude difference of ulnar stimulation between elbow and wrist was recorded from FDI and when median was stimulated in wrist whereas recording from FDI a small positive wave record from volume conduction of nearby muscles; (3) during stimulating ulnar nerve and recording from ADM, more than 20% of amplitude decline was detected between elbow and wrist; then median was stimulated in antecubital fossa whereas recording from ADM; provided that MGA was present, the amplitude difference of ulnar stimulation between elbow and wrist was recorded from ADM and when median was stimulated in wrist whereas recording from ADM, a small positive wave record from volume conduction of nearby muscles; (4) during stimulation of median at wrist and recording from Thenar muscle, the amplitude in proximal was higher than distal. By above mentioned criteria, conditions 2, 3, and 4 were regarded as presence of MGA.

In this research, our study population consists of 90 individuals including 59 women (65.6%), 31 men (34.4%), with an average age range of 40.72 ± 11.69 years and number of subjects with anastomosis was 13 cases. Therefore, the anastomosis frequency was 14.4%. Interpreting the results according to the number of hands for anastomosis, 15 hands (6 cases had the left hands and 5 cases had the right hands and 2 cases had the both hands) had anastomosis and the frequency of anastomosis was 8.33% for all hands. The types of anastomosis for these 15 hands, was as follows: 77 cases had normal first condition (85.6%), 9 cases had the second condition (10%) end to FDI, 2 cases had third condition (2.2%) end to ADM and 2 cases had fourth condition (2.2%) end to Thenar muscles. 

Our results are similar to other findings from Iran that estimated the prevalence of MGA as 10.3%.^2^ In the study was performed by medical students, the prevalence of MGA was around 20% indicating a high prevalence.^3^

In another study from Korea, MGA was found in 39.2% from 102 upper limbs. Among 12 instances of MGA between the branches innervating the ﬂexor digitorum profundus muscle, 8 anastomotic branches solely innervated the muscle without crossover from median to ulnar nerve.^4^

The major risk in not recognizing MGA is that of mistakenly interpreting the findings as ulnar conduction block. Apart from it, MGA has clinical significance for understanding median nerve lesion and carpel tunnel syndrome.

It appears from the present study that identification of MGA is extremely crucial before labeling the condition as ulnar neuropathy as the mode of treatment differs accordingly (former needs no treatment, whereas later needs it appropriately). Furthermore, we concluded that nerve conduction study is a reliable tool in diagnosis of MGA prevalence of, which we reported as 14.4% in EDX clinics of PM and R department Isfahan University of Medical Sciences, Iran. For future, the studies with larger samples may be required.

## Notes:

**How to cite this article: **Khosrawi S, Kianimehr L, Andalib S. The prevalence of Martin–Gruber anastomosis in Iranian subjects by electrodiagnostic criteria. Iran J Neurol 2015; 14(4): 231-2.
